# The feasibility and acceptability of using the Mother-Generated Index (MGI) as a Patient Reported Outcome Measure in a randomised controlled trial of maternity care

**DOI:** 10.1186/s12874-015-0092-0

**Published:** 2015-11-18

**Authors:** Andrew Symon, Soo Downe, Kenneth William Finlayson, Rebecca Knapp, Peter Diggle, SHIP trial team

**Affiliations:** Mother and Infant Research Unit, University of Dundee, 11 Airlie Place, Dundee, DD1 4HJ UK; Midwifery Studies, School of Health, Brook Building, University of Central Lancashire, Preston, PR1 2HE UK; School of Health, Brook Building, University of Central Lancashire, Preston, PR1 2HE UK; Sharoe Green Unit, Lancashire Teaching Hospitals NHS Foundation Trust, Preston, PR2 9HT UK; Lancaster Medical School, Lancaster University, Lancaster, LA1 4YB UK

**Keywords:** Patient-reported outcome measure, Randomised controlled trial, Quality of life, Pregnancy, Antenatal, Postnatal, Feasibility, Acceptability

## Abstract

**Background:**

Using patient-reported outcome measures (PROMs) to assess Quality of Life (QoL) is well established, but commonly-used PROM item-sets do not necessarily capture what all respondents consider important. Measuring complex constructs is particularly difficult in randomised controlled trials (RCTs). The Mother-Generated Index (MGI) is a validated antenatal and postnatal QoL instrument in which the variables and scores are completely respondent-driven. This paper reports on the feasibility and acceptability of the MGI in an RCT, and compares the resulting variables and QoL scores with more commonly used instruments.

**Methods:**

The single-page MGI was included at the end of a ten page questionnaire pack and posted to the RCT participants at baseline (28–32 weeks’ gestation) and follow-up (six weeks postnatal). Feasibility and acceptability were assessed by ease of administration, data entry and completion rates. Variables cited by women were analysed thematically. MGI QoL scores were compared with outcomes from the EQ-5D-3 L; Edinburgh Postnatal Depression Scale; Satisfaction With Life Scale; and State Trait Anxiety Inventory.

**Results:**

Six hundred and seventy eight pregnant women returned the pack at baseline; 668 completed the MGI (98.5 %); 383/400 returns at follow up included a completed MGI (95.7 %). Quantitative data were scanned into SPSS using a standard data scanning system, and were largely error-free; qualitative data were entered manually. The variables recorded by participants on the MGI forms incorporated many of those in the comparison instruments, and other outcomes commonly used in intrapartum trials, but they also revealed a wider range of issues affecting their quality of life. These included financial and work-related worries; moving house; and concerns over family illness and pets. The MGI scores demonstrated low-to-moderate correlation with other tools (all r values *p* < .01).

**Conclusions:**

Without face-to-face explanation and at the end of a long questionnaire, the MGI was feasible to use, and acceptable to RCT participants. It allowed individual participants to include issues that were important to them, but which are not well captured by existing tools. The MGI unites the explanatory power of qualitative research with the comparative power of quantitative designs, is inexpensive to administer, and requires minimal linguistic and conceptual translation.

**Trial registration:**

ISRCTN27575146 (date assigned 23 March 2011)

## Background

In terms of health care research, and especially randomised trials of effectiveness, death and serious morbidity tend to be the default primary outcomes. It is clearly useful to have common outcome measures so that data and results can be compared, and the COMET initiative [1] proposes the collection of core outcome sets within effectiveness trials. Within maternity care there is also a move to encourage the use of ‘core’ outcome measures [2]. Nevertheless, studies have tended to cast their net widely when deciding what to measure. Smith et al. [3] recent systematic review of Cochrane reviews concerning intrapartum studies identified 16 salutogenic (positive, health generating) outcomes and 49 outcomes focused on pathological phenomenon. A further recent study in the area of preterm birth found that 72 outcome measures had been used across 103 studies [4]. This divergence has led to a call for the development of core outcome sets for research across women’s health [2]. The proponents of this call recognise the need to include the perspectives of the women who use these services.

The attempt to be inclusive and comprehensive is welcome, but unlikely to be straightforward when those designing and funding RCTs tend to prioritise serious but rare outcomes (such as mortality) over more complex measures of experience and longer-term wellbeing that might apply to more of those in the general population—especially in maternity care where most women and babies are healthy. One solution has been the inclusion of patient-reported outcome measures (PROMs) to assess Quality of Life (QoL) [5–7] [8]. PROMs were originally developed to gauge the effectiveness of certain surgical procedures [6]. More recently, they have been expanded to a range of disciplines, in an attempt to “*seek to ascertain patients’ views of their symptoms, their functional status, and their health-related quality of life”* [9].

Despite the initial promise, it has been acknowledged that the wide range of professionally-derived proformas that are used to capture PROMs do not capture all the factors that matter to health service users [6]. It is difficult to square the need for an agreed and parsimonious core set for populations with particular health care needs, while still capturing the large variation between individuals within that population. The ideal instrument would encompass the specific needs, wishes, and priorities of each individual while at the same time providing a valid and reliable objective measurement that is comparable across populations and between studies. A validated QoL tool in which the variables and scores are completely respondent driven, but which could also provide a numeric QoL score would have utility not only in the assessment of clinical practice, but also as a key outcome measure in RCTs. This paper reports on the feasibility and acceptability of using one such tool, the Mother-Generated Index, in a trial context, and compares the resulting variables and QoL scores with more commonly used instruments.

### The Mother-Generated Index (MGI)

The MGI was developed from the Patient-Generated Index (PGI) [10] which has been used extensively, but never in the context of an RCT [11]. The PGI was the first attempt to develop a tool responsive to individual patients’ particular experiences and concerns, and which also converted those highly personal issues to a single Quality of Life (QoL) score that could be compared across populations. The MGI, in allowing individual respondents to identify and then score and rank the most important areas of their life, encourages this subjective evaluation.

The MGI is a one-page three-step questionnaire which generates a QoL score based on a list of variables that the mother herself identifies as being important in the context of the maternity care practice/intervention under examination. Because the variables the mother can record are not pre-specified, the MGI does not lend itself to a standard psychometric analysis [12], yet it has shown good face, criterion and construct validity [13], and its feasibility, acceptibilty, reliability and validity as a measure of maternity care in general have been established in observational and before-and-after studies within several linguistic and cultural groups in nine countries to date, and in both the antenatal and postnatal periods [14–17]. In all the studies to date participants were introduced to it during face to face interviews, although one postal follow-up has also been conducted [18].

**Fig. 1:**
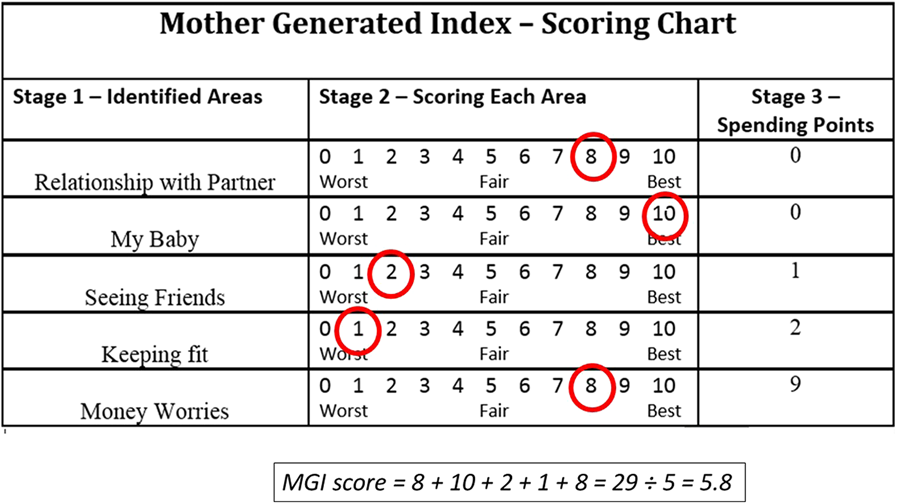
Example of completed MGI form

In Step 1 of the MGI the woman records her subjective description of what is important to her. Based on how she has been affected over the previous month by these clinical, emotional, cultural and psycho-social concerns she then scores each area in Step 2 to produce a primary index of quality of life (range 0 [worst] to 10 [optimum]). This score, the average of the individual variable scores, can be compared objectively across whole populations. In Step 3 she allocates ‘spending points’ to indicate the relative importance of the areas she has cited. Figure 1 provides an example of a completed MGI form.

We could not find any report of a completely respondent-driven PROM such as this being used within a trials context. We decided to include the MGI in a pre-trial pilot of the SHIP trial [19]. Positive results from this pilot convinced us to include it in the main trial. We therefore set out to examine whether the MGI was feasible and acceptable as a respondent-driven PROM within a randomised controlled trial. We believe this to be the first time that an individually patient-generated postal PROM has been tested within the context of an RCT.

## The context

The SHIP (Self-Hypnosis for Intrapartum Pain) trial took place in seven sites across three NHS Trusts in the northwest of England, including a range of birth settings (free standing midwife led units, an alongside midwife led units, and three hospitals with birth rates of 10,300, 6,900, and 4,500 in 2013). The Trusts covered both rural and urban populations with a range of socio-demographic profiles. The trial assessed the effect of a group-based antenatal self-hypnosis education programme on rates of epidural use in labour. Secondary outcomes included several measures of wellbeing and satisfaction using standard data collection tools. The 680 participants were nulliparous women not planning elective caesarean, without medication for hypertension and without psychological illness. A ten-page questionnaire pack was sent by post to women in both the intervention and control groups at baseline (28–32 weeks), 36 weeks gestation, two weeks postnatally, and six weeks postnatally. The MGI was included at the end of the pack at baseline and six weeks postnatal.

## Methods

Assessing feasibility and acceptibility of the MGI entailed an examination of how well the tool could be integrated into the questionnaire pack, how easily the data items could be scanned with a standard data scanning system, an assessment of the percentage of participants who attempted the MGI even though it was at the end of a long questionniare pack, and the average percentage of elements of the instrument completed, and completed correctly at both time points. The validity of the MGI had already been establised in a range of cultural contexts, so formal validation was not required. However, a comparision was made between the MGI QoL scores at both time points, and the same woman’s scores for several psychometric tools commonly used in maternity care service development and research. Finally, to test the hypothesis that a PROM based on what was important to each individual trial participant would capture different variables and concepts than commonly used tools in maternity care trials, we examined these data thematically, and compared them to the variables in the comparator tools in the SHIP trial, and to the outcomes that were recorded in a review of Cochrane reviews of intrapartum RCTs (Smith et al. as above [3]).

### Comparator instruments

Four of the measures in the questionnaire pack were analysed as comparators for the MGI. The EQ-5D-3L [20] includes questions regarding mobility, self-care, usual activities, pain/discomfort and anxiety/depression. The Edinburgh Postnatal Depression Scale [21] is a widely-used ten-question screening instrument that is validated for both antenatal and postnatal use. The Satisfaction With Life Scale [SWLS] [22] is a five-item instrument measuring global cognitive judgements about life satisfaction. The questions relate to whether life is ideal or not, whether conditions are good, whether the respondent has achieved most of the things they want to achieve or would change things if they had the chance. The short version of the State Trait Anxiety Inventory [STAI] [23], a widely-used tool which measures anxiety, is said to produce similar scores to the full version. Between them, these instruments aimed to measure health status, satisfaction with life, anxiety and depression.

### Data entry

As part of the whole questionnaire pack, the completed MGI results were scanned directly into the study database for each respondent, and this was overseen by the Clinical Trials Unit (CTU) associated with the study. The scanning software identified numerical responses. Narrative responses were entered into the database by hand.

### Analysis

Regular quality checks within the CTU tracked data entry errors or unusual responses. Quantitative data from the questionnaires were analysed in SPSS. Simple percentages were used to determine completion rates. Simple correlations using Pearson’s test were used to compare the MGI QoL score and those on the comparator tools.

Each MGI form was also evaluated by a thematic analysis of the variables cited in Step 1; this was done independently by paired members of the team. Disagreements over themes were resolved at a team meeting. KF, AS and RK independently generated themes from the narrative data and matched the numeric data with the emergent themes to produce frequencies as well as the primary and secondary indices.

We then compared this analysis with the components of these responses to the comparator tools and with the outcomes identified by Smith et al. [3] in their systematic review of outcomes in the Cochrane Pregnancy and Childbirth database.

Ethics approval was granted by an NHS IRAS Ethics Committee and by the University of Central Lancashire, Faculty of Health Ethics Committee. Formal written consent was obtained from all participants before recruitment to the study. This article presents independent research funded by the National Institute for Health Research (NIHR) under its Research for Patient Benefit (RfPB) Programme (Grant Reference Number PB-PG-0808-16234). The views expressed are those of the author(s) and not necessarily those of the NHS, the NIHR or the Department of Health.

## Results

The baseline survey at 28–32 weeks gestation was completed by 678/680 women (99.7 %), and 400 of these 678 completed the postal follow-up survey at 6 weeks (59 %). Their socio-demographic data at baseline are shown in Table 1. The socio-demographic characteristics of those at follow-up were broadly similar across all variables, the only exception being that those lost to attrition had slightly lower educational attainment (*χ*^2^ = 8.54; *p* < .05).

**Table 1:** Socio-demographic characteristics of participants at baseline

Variable	Total	Intervention	Control				
	N	n	Mean	SD	n	Mean	SD
Age	672	337	28.4	5.5	335	28.5	5.2
Gestation at randomization	669	335	27.8	1.0	334	27.8	1.1
	N	n	n of event	%	n	n of event	%
Education (% GCSE or below)	665	333	70	21.0	332	54	16.3
Ethnicity (% White)	670	336	320	95.2	334	303	90.7
BMI > 40 (% at booking)	672	337	8	2.4	335	9	2.7
Income (% < £24,000 p/a)	652	324	99	30.6	328	89	27.1
Birth partner identified (% yes)	669	335	331	98.8	334	334	100.0
Type of Maternity Care (% midwife led)	655	327	287	87.8	328	288	87.8

### Feasibility of using the MGI within an RCT

The MGI took up one page in the ten page questionniare pack and was easily integrated into theis. The Clinical Trials Unit reported that the scoring for Steps 2 and 3 of the MGI was picked up well by the scanning system they used, and that the data were largely free of errors (see below for details). However, the narrative data in the free text boxes (which described the variables of interest to each woman) had to be checked and entered by hand.

### Acceptability/ease of use

The percentage of questionnaire respondents who recorded at least some response on the MGI component was 98.5 % (668/678) at baseline (338/342) [98.8 %] intervention group and 330/336 [98.2 %] control group) and 95.7 % (*n* = 383/400) at 6 weeks postnatal (200/207 [96.6 %] intervention group and 183/193 [94.8 %] control group). At baseline, 94.8 % (633/668) of respondents listed the maximum number of five issues in Step 1 of the MGI with 20 citing four issues, nine citing three, two citing two and four citing one. Completion of the MGI Step 1 was also high at six weeks postnatally: 91.6 % (351/383) of participants listed five issues, 20 cited four, six wrote three, four listed two and two cited one issue. All participants who completed a postnatal MGI form had also completed the baseline MGI. All participants at both time points were able to allocate a score at Step 2 of the MGI but 21.4 % (142/662) of participants at baseline and 27.6 % (103/383) of women six weeks postnatally did not allocate the Step 3 points correctly. These inaccuracies were largely due to using more or fewer than the 12 permitted points or not allocating any points at all. As the calculation of the QoL measure does not depend on Step 3 of the MGI, this did not affect the capacity of the instrument to measure QoL quantiatively. With face-to-face surveys any difficulties with form completion can be overcome; improving the instructions for Step 3 completion in future postal surveys should mitigate this problem.

### Comparison with other instruments

The descriptive statistics for the various questionnaires are shown in Table 2. At baseline and postnatal follow-up the MGI showed statistically significant low-to-moderate correlations with all of the standard questionnaires. The lowest correlations were recorded against the EQ-5D. At baseline 64 % (426/662) women scored ‘1’ (indicating perfect quality of life) on the EQ-5D; at follow-up (6 weeks postnatal) 60 % did so (239/385). This suggests that the EQ-5D has very low discriminatory power for antenatal and postnatal women, who are largely healthy.

**Table 2:** Standard psychometric measures: descriptive statistics and correlation with MGI

						Correlation with MGI	
		Mean	SD	Range	IQR	r	p
MGI	Baseline	7.6	1.51	0–10	1.80	n/a	
	6 weeks PN	7.6	1.48	0–10	1.65	n/a	
EQ-5D-3L	Baseline	.92	0.12	0.13–1	0.20	.225	*p* < .01
	6 weeks PN	.91	0.15	−.594–1	0.15	.226	*p* < .01
EPDS	Baseline	6.4	4.53	0–26	6.00	−.350	*p* < .01
	6 weeks PN	4.9	4.41	0–22	6.00	−.395	*p* < .01
SWLS	Baseline	28.1	4.88	9–35	5.00	.464	*p* < .01
	6 weeks PN	29.3	4.57	10–35	6.00	.334	*p* < .01
STAI	Baseline	10.2	3.52	6–22	5.00	−.255	*p* < .01
	6 weeks PN	9.3	3.42	0–23	5.00	−.306	*p* < .01

Key

*IQR* Interquartile Range, *EQ-5D-3L* Euroqol 5D [3L version], *EPDS* Edinburgh Postnatal Depression Scale, *SWLS* Satisfaction With Life Scale, *STAI* State Trait Anxiety Inventory, *6 weeks PN* 6 weeks postnatal

### Comparing the reported issues in the MGI with those in existing standard instruments and outcomes reported in a systematic review of reviews of intrapartum interventions

At baseline the 668 respondents cited a total of 3,280 comments in the MGI. In addition, both Step 2 scores (reflecting how the woman had been affected by the cited issue over the previous month; possible range 0-10) and Step 3 scores (her relative ranking of importance; possible range 0–12) varied considerably (Table 3).

**Table 3:** Frequency of cited comments at baseline (27 weeks gestation), with associated MGI scores

		Step 2 scores			Step 3 scores		
	Comments	Mean	SD	Range	Mean	SD	Range
Partner	653	8.3	1.9	0–10	2.4	2.2	0–12
Extended family	550	8.2	2.2	0–10	2.5	2.4	0–12
Career/work	468	6.3	2.2	0–10	3.2	2.5	0–12
Health/Wellbeing (Self)	314	7.5	2.4	0–10	2.9	2.2	0–12
Friends	277	7.6	2.0	0–10	2.5	2.0	0–12
House/Home issues	219	8.4	2.3	1–10	2.6	2.3	0–12
Money	195	5.6	2.1	0–10	3.7	2.4	0–12
Baby	186	8.7	1.8	0–10	3.1	2.4	0–12
Transition: Preparation & Planning	147	8.2	1.8	3–10	2.9	2.3	0–12
Becoming a Mother/Family	65	9.0	1.7	2–10	2.4	2.3	0–12
Education	39	6.6	2.4	2–10	2.8	2.0	0–10
Health/Wellbeing (Others)	34	6.2	3.0	0–10	5.0	3.8	1–12
Animals/Pets	33	7.6	3.2	0–10	2.9	3.2	0–12
Labour & Childbirth	22	4.7	3.1	1–10	4.2	3.2	1–12
Faith/religion	13	8.5	1.9	5–10	5.2	3.3	2–12
Other	65	6.6	2.7	0–10	3.3	3.0	0–12

The most commonly cited issues related to the woman’s partner, extended family, career/work concerns, and her own health and wellbeing. The woman’s wider social circle, issues regarding house and home, money, and the anticipated baby, were important too, although several other themes also emerged such as the transition to parenthood and concern over illness within the family. These themes partially echoed those variables included in standard item-sets and Smith et al. [3] review of Cochrane reviews, but there were many which stood apart from these ‘standard’ outcomes.

Women who cited career and work issues gave them varying scores. Low-scoring comments (“Completing workload before I leave”, “Stress at work” and “Returning to work after maternity leave”) were balanced by more positively worded ones (“Good working relationships”, and “Being in a stable job and financially secure”). Money issues were commonly expressed, and these were mostly negative (“Feeling isolated and lonely due to money”, and “Not having enough money to get married or get a mortgage”). Many found themselves wanting or having to move house (“Finding a house”; “Getting a fixed address”) or preparing the home (“Getting the house ready for baby”). Less expected areas included concern over a friend whose baby had been born disabled, references to animals (“My pet cat being stressed at changes”; “My dog has cancer”), to hobbies and to faith and religion.

The 1,861 comments cited by 383 mothers at postnatal follow-up were categorised thematically, and the Step 2 and Step 3 scores analysed as before (Table 4).

**Table 4:** Frequency of cited comments at baseline (6 weeks post-natal), with associated MGI scores

		Step 2 scores			Step 3 scores		
	Comments	Mean	SD	Range	Mean	SD	Range
Partner	364	8.4	1.8	0–10	2.7	2.0	0–12
Baby	296	9.0	1.7	2–10	2.4	2.4	0–12
Extended Family	267	8.5	1.7	0–10	2.2	2.2	0–10
Friends	189	7.1	2.0	2–10	2.5	1.7	0–9
Career/Work	144	5.8	2.4	0–10	3.1	2.4	0–12
Becoming a Mother/Family	142	8.9	1.4	3–10	2.6	2.1	0–11
Health/Wellbeing (Self)	137	6.2	2.5	0–10	3.5	2.4	0–12
House/Home Issues	124	6.6	2.1	0–10	3.1	2.5	0–12
Money	102	5.7	2.1	1–10	4.1	2.5	0–12
Transition: Preparation & Planning	23	6.5	2.4	1–10	3.1	1.8	1–6
Animals/Pets	16	7.0	2.9	0–10	3.3	2.6	0–9
Education	12	5.2	2.7	1–10	5.2	3.7	1–12
Health/Wellbeing (Others)	7	4.9	2.2	2–8	4.7	3.7	1–12
Faith/Religion	5	8.4	1.4	7–10	3.8	1.9	2–7
Other	33	6.2	3.2	0–10	3.4	2.8	1–12

As with the baseline comments these varied considerably. Most postnatal comments fell into fairly predictable categories, reflecting outcomes measured in other studies: joy or concern about the baby, relationship with partner and parents, personal health and wellbeing, and finding (or not finding) a routine or new social circle. This compares with Smith et al. [3] review which identified (albeit in very small numbers) ‘Positive relationship with infant’, ‘Wellbeing’ (her own and the partner’s), and ‘Views’ (again her own and her partner’s).

However, the MGI identified some complexity in these issues, reflecting personal difficulties or sadness (“Relationship with baby’s father”; “Best friends leaving the area”; “Remembering my dad”). Several commented on the lack of ‘Me time’ (i.e. time to herself). While some comments expressed the happiness of the new or changing role (“Being a good mum”; “Making new friends through baby groups”; “Being able to socialise and drink again”), others revealed traumas (“Feeling out of control [unable to plan day/night due to feeding demands]”). One mother who cited “Post-traumatic stress brought on by nightmares due to care whilst in labour” did indeed have a higher than average STAI score. For some, other issues were important: one referred to starting her own business, and another to having to learn a script for a play. Religion (“Faith in God – Christianity”) and animals (“Exercising and bonding with dog”; “Making time for my two cats”; “My horses”) featured again.

## Discussion

This paper evaluates the incorporation of the MGI, a respondent-driven Patient-Reported Outcome Measure, as a postal QoL instrument within a randomised controlled trial of self-hypnosis for labour pain amongst nulliparous women who were unselected for risk. We found the incorporation of the MGI to be feasible, acceptable and informative. Although the women had not seen the MGI beforehand, completion rates were encouragingly high at both baseline and follow-up, despite the fact that it was the last questionnaire within a ten-page study pack, and presumably the last to be completed. The high response rate and the generally accurate form completion indicate that this respondent-driven instrument is an acceptable way of determining quality of life as a trial outcome, but based authentically on what matters to each individual participant. Although the MGI only had low to moderate correlation with a range of instruments that are frequently used in maternity trials, we would suggest that this might be because the MGI is better at tapping issues that really matter to the individuals in the study, in contrast to the fixed variable sets in the existing tools. While some of the themes identified at baseline and follow-up were to be expected (‘partner’, ‘baby’, ‘extended family’ ‘work’, ‘house and home’), others were less predictable. The MGI allowed the women to raise issues like education, the health and wellbeing of significant others, financial issues and even pets and religion. In addition, they were able to score and rank these. These are aspects of their lives that most standard tools will not cover.

An additional added value of the MGI is that it requires minimal conceptual and linguistic translation if it is just used as a measure of QoL. In this case, the variables recorded by the participants do not need to be translated, as what is of interest is the final numeric score. The instructions are brief and easily translated. The successful use of the MGI in a wide range of context, including India [15] Germany [16] Poland [24] and Iran [14] demonstrates its utility as an outcome measure that can transcend language and cultural barriers with relative ease. This is a significant advantage for international multicentre studies and also for studies in a single country that include a wide range of cultural and language groups, as is often the case for those accessing maternity care.

### Strengths and weaknesses of the study

The main strength of this study is that it was undertaken alongside a robust clinical trial with a very high response rate at baseline. While the trial did experience much lower responses at the six week follow up, this was no different for the MGI than for the other tools in the questionnaire package, and, at both time points very high percentages of respondents completed the MGI. Limitations included variation in handwriting in self-completed forms, which made electronic scanning of free text entries problematic. As a consequence, manual entry of comments was required. The fact that around one in five respondents did not complete Step 3 of the instrument correctly suggests that another limitation was a lack of clear instructions as to how to undertake this element of the tool. The order in which the various instruments were offered in the questionnaire pack may have affected what was cited in the MGI. Given that the tool depends on women to report what matters to them, it may be best to place it at the beginning of the pack, so that the items in the other instruments do not influence maternal responses. In fact, however, while some of the comments identified by participants reflected domains present in standard psychometric tools, many did not, so order of questionnaire completion did not seem to influence the MGI responses in this study.

### Strengths and weaknesses in relation to other studies

To our knowledge no previous RCT has included an individualised respondent-driven PROM such as this, although many studies have used health status or specific quality of life instruments. Basing quality of life assessment on the areas of life which the mother considers to be those most important to her avoids the pitfalls of a ‘top-down’ instrument which, however well prepared, may not reflect the woman’s current concerns [25]. Most of the women in this study cited partner or other family members as important considerations, yet, these variables are not usually included in the assessment of intrapartum outcomes in controlled trials in maternity care [3] and nor are they an element of the EQ-5D, STAI short version or the SWLS. The EPDS, a screening tool for maternal psychological wellbeing, does not even mention the baby, a factor in the new mother’s life that was worthy of note to almost all the mothers surveyed here. Other factors not covered by the standard tools but recorded as important by the respondents to this study included financial worries, the stress of moving house, anxiety concerning work or education, and the importance of friends.

Satisfaction is commonly used as an indicator of patient experience, but this approach has been criticised because of a lack of standardisation and of reliability data [26]. The link between patient satisfaction and the health care system or health care quality and outcomes has been claimed to be unclear and even tenuous [27, 28]. In condition-specific cases a targeted quality of life evaluation may be required (*e.g.* [29]). However, the experience of pregnancy and new motherhood cannot be described as neatly as with a specific medical condition, and the MGI allows a holistic approach to be adopted that goes beyond satisfaction measures. The MGI has been used in the UK, Poland, Portugal, Brazil, Iran, China, India Germany and Switzerland [14–17, 24]. Given the very limited textual and conceptual translation that is needed for the data collection tool, the MGI in most cases is easily transferable into the full range of cultural and linguistic contexts in which maternity care is provided. Other tools, by contrast, often require sophisticated and expensive conceptual and linguistic fine-tuning to fit each context in which they are used.

Many tools are available in a range wide of languages, but this ease of use has potential pitfalls: if researchers assume they are measuring one construct when they are measuring another, then false inferences may be made. The EQ-5D, for example, has been referred to as a ‘quality of life’ tool [30, 31], although properly speaking it measures health status. Choice of tool must be informed by a clear understanding of what is being assessed.

### Implications for clinicians and policymakers

The MGI is already recognised as a valuable tool for assessing women’s views and experiences of maternity care in general. Extending it as a key outcome measure for clinical trials would enable researchers to capture complex and nuanced variations in what matters to women. A QoL tool that allows scores to be compared numerically between intervention and control groups, and which also allows for more nuanced analysis of the issues that affected women in relation to the trial, adds a valuable extra layer of understanding. This can also be used as a basis for understanding the contextual factors that might influence the success or failure of the introduction of complex interventions into trials, opening up what has been called the ‘black box’ of the mechanisms of effect in such interventions [32]. These added insights might also enable more efficient transfer of knowledge from research to practice, as they could frame the way in which an intervention should be implemented if it is successful.

### Unanswered questions and future research

The potential for the MGI to explain mechanisms of effect of interventions in trials, and for this to inform roll-out, needs to be assessed in future research. We would recommend that the MGI is tested further in future controlled studies of maternity care, and that the Patient-Generated Index is also considered for use in controlled trials in studies of health care in general. Apart from one postal follow-up, prior to the SHIP Trial the MGI had only been completed under the supervision or guidance of a researcher. However, given the excellent completion rates in this study there would appear to be scope to test the distribution of the tool on a variety of platforms including internet and mobile devices.

The timing of the MGI’s application is an issue: it has been used in various studies from the late pregnancy to the immediate postnatal period and up to eight months following the baby’s birth. Since quality of life is dynamic, it will clearly identify different issues at different times. Large-scale longitudinal studies, including RCTs, would allow for the tracking of changing issues of concern and possible changing quality of life scores as well. Given the prominence of comments relating to partners in the SHIP study, and the known importance of social support during and after pregnancy [33, 34], it would be instructive to examine the quality of life of women and partners as dyads.

### Conclusion

With greater demographic and workforce mobility there is increasing diversity within local populations; this complexity is a factor of modern contemporary health care and research. While Black [9] draws a distinction between PROMs and patient-reported experience measures (PREMs), in practice it may be difficult to separate out a patient’s perceptions of a clinical condition and their reported experience of clinical care related to this. We have demonstrated that the MGI is a feasible, acceptable, and value-added quality of life measure for assessment of outcome at two points in time in trials of maternity care. It unites the explanatory power of qualitative research with the comparative power of quantitative designs, is inexpensive to administer (and would be even on a range of platforms) and, based on previous studies, it is easily translated to a range of cultural and linguistic contexts.

## Abbreviations

CTU: Clinical Trials Unit
EPDS: Edinburgh Postnatal Depression Scale
EQ-5D-3L: Euroqol 5D [3 L version]
IQR: Interquartile Range
MGI: Mother-Generated Index
PGI: Patient-Generated Index
PREM: Patient-Reported Experience Measure
PROM: Patient-Reported Outcome Measure
QoL: Quality of Life
RCT: Randomised Controlled Trial
SHIP: Self-Hypnosis for Intrapartum Pain management trial
STAI: State Trait Anxiety Inventory
SWLS: Satisfaction With Life Scale
